# Pediatric First-Degree Burn Management With Honey and 1% Silver Sulfadiazine (Ag-SD): Comparison and Contrast

**DOI:** 10.7759/cureus.32842

**Published:** 2022-12-22

**Authors:** Md. Moniruzzaman, Abdur Rahed Khan, Md. Ahsanul Haq, Rawshon Ara Naznin, Mainul Haque

**Affiliations:** 1 Pediatric Surgery, 250 Bedded General Hospital, Kushtia, BGD; 2 Pediatric Surgery, Dhaka Shishu Hospital, Dhaka, BGD; 3 Bio-Statistics, Infectious Diseases Division, icddr,b, Dhaka, BGD; 4 Anatomy, TMSS Medical College, Bogura, BGD; 5 Pharmacology and Therapeutics, National Defence University of Malaysia, Kuala Lumpur, MYS

**Keywords:** bangladesh, postburn contracture, scar, cooking fire, hot water, honey, silver sulphadiazine, wound healing, children, burn

## Abstract

Background

The cardinal area of managing fire wounds is guided by adequately evaluating the burn-induced lesion's profundity and size. Superficial second-degree burns are often treated through daily reinstating with fresh sterile bandaging with appropriate topical antimicrobials to allow rapid spontaneous epithelialization. Around the world, a wide variety of substances are used to treat these wounds, from honey to synthetic biological dressings.

Objective

This study intended to determine honey's therapeutic potential compared with 1% silver sulfadiazine (Ag-SD) in arsenal-caused contusion medicament fulfillment.

Methods

A total of 70 cases were evaluated in this research work after fulfilling the required selection criteria during the study period of January 2014 to December 2014 and January 2017 to December 2017. Purposive selection criteria were adopted in the study to select research patients. The patients in Group-1 (n = 35) relied on honey as medication, while patients in Group-2 (n = 35) relied on 1% Ag-SD.

Results

In Group-1, exudation (68.4%) and sloughing (82.9%) were substantially reduced by Days 3 and 5 of therapeutic intervention, respectively. However, in Group-2, a reduction of exudation (17.1%) and sloughing (22.9%) occurred after Days 3 and 5 of treatment, respectively. Completion of the epithelialization process was observed among Group-1 and Group-2 cases. It was detected after Days 7 and 10 of treatment at 36.3% and 77% (Group-1) and 27% and 67% (Group-2), respectively. Around 3 ml of 1% honey was required per body surface area per dressing in Group-1. On the other hand, in Group-2, 2 gm Ag-SD was needed per body surface area per dressing.

Conclusion

Patients treated with honey found better clinical outcomes in managing superficial partial-thickness burns.

## Introduction

A burn is a heat-induced acute trauma [1]. It is often brought by chemical, electrical, friction, or radiation, physical, and chemical (organic or inorganic) agents [2]. Burn injury uniformly involves local and systemic adverse impacts on any living creature, including humans, with short- and long-term consequences [3,4]. Severe burns have been observed to upset cardio-vascular physiology resulting in hypovolemic and distributive shock [1]. It also involves the immune and metabolic systems and causes disastrous inflammatory retaliation to catalyze diversiform organ failure and promote sepsis [1,5,6]. Burn causes not only bodily damage but also causes physical limitation, with promotes cosmetic issues. Nevertheless, it also damages psychological and emotional well-being, impairing the patient's future quality of life [4,7,8]. Thermal burn wounds are common among pediatric cases and a significant cause of childhood trauma [9-11]. Burn management frequently needs multidisciplinary care [12,13]. It often causes fatal outcomes, especially among children, everlasting indelible defacement, mutilation, and anatomical and physiological malfunction [4, 14-17].

Categories of burn

Skin is the largest organ system of our body, and its weight is 16%, which is about one-seventh of total body weight [18-20]. Skin acts as a shielding fence, managing evaporation, controlling temperature, expelling waste products, and carrying sensations such as pain, touch, temperature, and pressure [21]. Burns encompass a range of intensity of injury determined by the profundity of the maim and the quantity or percentage of the burn of the whole body [22]. Burn has been categorized as superficial (first degree), partial thickness (second degree), and total thickness (third degree), involving just the epidermal layer of the skin, damaging to deeper structures within the skin, and all layers of the skin, respectively [23-25]. It has been reported that burns have the possibility to damage structures beneath the skin and deeper tissues. Thereafter, this clinical condition is called ﻿total thickness burn, which can entangle more inner tissues (fourth degree) [21]. Similarly, superficial or first-degree burns only include the epidermal layer of skin [26,27]. Partial thickness burns are often defined when 10% of the total body surface area (TBSA) is affected and commonly affect people aged 10-50 years [22]. A third-degree burn indicates a full-thickness epidermis and dermis, affecting over 15% of the TBSA [22].

Burn-related morbidity and mortality

It has been estimated that 180,000 patients have fatal outcomes globally every year due to burns [28]. Additionally, there is high-rate morbidity, prolonged hospital stays, and management of burn involves financial burden over healthcare. The treatment of burns increases substantial impediments to the healthcare system at the public and community levels around the globe, especially among low-income countries [29]. The number of global deaths due to burning was recorded as 120,000-265,000 per year, most importantly of which occurred in low- and middle-income countries (LMICs) [30,31]. Multiple studies have reported that the overall mortality rate in Bangladesh is 2-2.2 per 100,000 people per year [25,26]. The fatal clinical outcome has been more among female victims [31,32]. Additionally, burn-related injuries in Egypt, Colombia, Pakistan, and Bangladesh give rise to 18% of permanent disorders and special needs [33]. In low-income countries, children below 5 to 6 years are at maximum risk of burn-related trauma as they have thinner skin [34,35]. Flame and scald burns remain the primary contributors to burn injuries among infants, children, and adolescents [36-38]. Subsequently, burns are the most prevalent and calamitous type of wound that requires surgical management. Nevertheless, this global public health issue has been reported to be preventable [25,39-42].

Burn and infection

One study reported that the infection is a typical stumbling block in the therapeutic intervention of post-burn wounds [43]. *Providencia rettgeri* is a gram-negative bacillus. *P. rettgeri* is an atypical pathogenic microbe that hardly ever causes wound infections.* P. rettgeri* often infect burn wound and causes substantial morbidities. Furthermore, drug resistance towards *P. rettgeri* further complicates burn infection. Additionally, antimicrobial resistance raises treatment difficulty, especially in low-budget healthcare settings [43]. Additionally, one more study revealed that 75% of fatal outcomes result from sepsis from a burn-wound infection [44]. Resistance microbes infect burn wounds, causing sepsis, often hindering the therapeutic process [45]. Consequently, worldwide infection of burn wounds endures the principal mainspring of high-level morbidity and mortality [1]. Multiple studies also revealed that the death rate is as high as 51% [14,46-48]. One Cochrane study reported that 75% of burn cases died from life-threatening microbial infection after a preliminary emergency procedure [49]. Most dermatological burns are minor and treated with domiciliary care [50, 51]. Minor skin burns are often managed at home with cold water and several over-the-counter burn creams and gels [52].

Burn and hospitalization

Patients with burns in either pediatric or adult cases require urgent hospitalization when affected areas cover 5-10% and 10-20% of TBSA, respectively [52]. Additionally, burns involving hands, feet, neck, face, airway, and perineum demand hospital care [52,53]. Furthermore, burn cases with diabetes, immunosuppressed circumstances, and of extreme age, either pediatric or geriatricindividuals require high-profile medical care [54-58]. 

Modalities of burn infection control

Commonly used antimicrobials in burns management include ﻿neosporin, polymyxin B, mafenide acetate, mupirocin, bacitracin, nystatin, nitrofurazone, etc. [59-62]. Additionally, silver derivative compounds are also utilized in burn infection management. Those compounds include Ag-SD, flammacerium, silver amniotic membrane, silver nitrate, acticoat 7, silver foams, aquacel-Ag, and silvercel [62-65]. Furthermore, iodine-based compounds have also been used in cadexomer iodine, povidone-iodine, repithel, liposomal iodine, and Iocide [62,66,67]. Several antimicrobial peptides, such as histone H1.2, ceragenins, demegel, defensins, cecropin B, rBPI, etc. have been reported to have beneficial effects regarding burn infection control [62,68,69]. It has been observed that multiple synthetic and herbal compounds have also been used in burn-related infection management. Those include papaya, honey, bicomponent triton tri-n-butyl phosphate (BCTP) nanoemulsion, acidified nitrite, chlorhexidine, mitogen-activated protein kinase (p38 MAPK) inhibitor, 1-ethyl-6-fluoro-1,4-dihydro-4-oxo-7(1-piperazinyl)-quinoline-carboxylic acid (FPQC), moist exposed burn ointment (MEBO), probiotics, lactobacillus, phage therapy, super oxidized water, essential oils [62,70-76]. 

Critical issues of burn treatment

A censorious segment of burn wound medical intervention is correcting fluid and electrolyte imbalance, nutritional intervention, organ support, wound care, and overall resuscitation [29,77-79]. Additionally, burn wound management requires rapid healing with minimum scarring [25,80-83]. Multiple procedures are advocated for therapeutic intervention of burn wounds [84-86]. Open or exposed, semi-closed, or complete bandaging burn wound procedure, with or without antibiotics, has been often practiced in many developing countries [87-89]. Burn patients are usually kept strictly under the mosquito net in ambulatory and hospitalized cases in many LMICs to prevent maggots’ formation [90,91]. The open approach line-up with desiccating up the burnt area in the earliest possible time, restoring the site, and rejuvenating beneath the withered crust [92]. Burn patients had a high possibility of developing infections. Thereby, infection control and management remain as centerpiece of therapeutic intervention [93,94]. Additionally, these patients required encouraging or fostering skin tissue reconstruction (epithelialization) activity [95]. Several modalities regarding infection control among burn cases have been practiced around the globe [96].

Silver sulfadiazine (Ag-SD) in burn infection control

It has been reported that long-standing burn patients often had fatal outcomes, including death (42-65%) because of infection [97-101]. Ag-SD is a pharmaceutical product often prescribed to stave off, control, and treat infectious disorders in burn lesions. It is a heavy metal dermatological medication with antimicrobial effects [59,102,103]. Ag-SD has been considered the gold standard for therapeutic intervention regarding infection control of burn wounds for topical, second- and third-degree burns [62,104,105]. Ag-SD (1%) topical formulation has been reported to possess potentially good efficacy with an admirable low adverse profile for superficial and partial thickness burn [106-108]. Ag-SD has been considered a potent antimicrobial medication and appraised as a conventional or typical strategy for burn-affected individuals [109]. Nonetheless, one meta-analysis revealed that new dressings for burn treatment containing silver or without silver have better efficacy than Ag-SD regarding wound alleviation. Additionally, dressings that do not contain silver had a lower possibility of developing an infection than with Ag-SD. Moreover, statistically significant disagreement was observed between Ag-SD and new silver materials containing dressings associated with averting infection [110]. The efficacy of Ag-SD regarding burn wound healing is solely dependent on its antimicrobial properties; thereby, Ag-SD has been utilized for decades [111, 112]. Multiple studies reported that several microbial agents resist Ag-SD [62,104,113].

Role of honey in the management of burn

Globally, multiple natural substances, traditionally used as medication, especially honey, have been used to aggrandize burn-induced trauma alleviating, especially for about 8,000 years [114-118]. Burn dressings containing honey minimize pain, decontaminate, disinfect burn lesions, and expedite the restorative and beneficial process [113,119]. It has been reported that honey's valuable contributions to burn are its anti-inflammatory, antioxidant, and anti-microbial properties that ensure the success of skin grafting and wound mitigating process [117,120, 121]. Honey comprises various subcategories of carbohydrates, lipids, amino acids, proteins, vitamins, and minerals that are predominant in burn-trauma curative effect and minimizes further injury throughout the dressing process [122]. Vitamin C, monophenolics, flavonoids, and polyphenolics like watery and lipotropic anti-oxidants are commonly available in honey. Additionally, aqueous and blacker honey contains more antioxidants, consequently, acts as an ideal natural oxidants antagonist [123-126]. Many research papers have postulated several mechanisms regarding honey's anti-inflammatory effect. Those are suppression of synthesis of nitric oxide and complement, impediment of macrophage activity, curbing and squashing of reactive oxygen species (ROS) by phagocytes, minimizing free radical formation reducing oxidative stress, and availability of apalbumina-1 in honey because this chemical moiety secreted by the honeybees. Apalbumina-1 is known to possess an immunostimulatory effect [127-130]. Antimicrobial properties of honey have been explained as it contains high sugar, which causes the osmotic effect. Thereby preventing the growth of microbes in the wound and enhancing the healing process [131-133]. Another study reported that honey produces antimicrobial effects through the enzymatic production of hydrogen peroxide. Although some medicinal honey shows an antimicrobial effect, even hydrogen peroxide activity is blocked [117,134]. However, honey’s medicinal properties differ in the country of origin, type of plant, and bees [135]. Besides honey’s antimicrobial effects, it has an antioxidant effect that neutralizes free radical formation inflammatory response because of burn [114,136]. Additionally, honey's hygroscopic effect, high viscosity, acidic pH, and hydrogen peroxide content produce comprehensive favorable results in burns therapeutics and care [114,119]. Multiple studies reported that honey’s hydrogen peroxide, also known as inhibin, is the principal component responsible for antimicrobial properties [135, 137]. The burn area required slightly wet burn surroundings for fast remedy. Honey effectively generates moist conditions and promotes ideal healing [114, 132]. Additionally, honey-induced damp conditions quickly reduce edema, and exudates, remove the infection, decreases inflammation, and freshen and sanitize burned wound area [132,138]. Furthermore, honey enhances the rapid re-epithelization process in burn-induced lesions called the epithelial-mesenchymal transition (EMT) and stimulates angiogenesis and the immune system [116,136,139-141].

Objective of the study

This study intended to assess the effectiveness of honey in comparison with 1% Ag-SD in burn wound management.

## Materials and methods

Study details

Study Design and Sample Selection

This was a prospective comparative study conducted in the burn and reconstructive surgery unit of Dhaka Shishu Hospital, Dhaka, Bangladesh. The age range was 12-60 months. 

Study Period, Sampling Method, and Sample Size

From January 2014 to December 2014 and January 2017 to December 2017, totaling two years. 70 patients were incorporated into this study after fulfilling the all-inclusive required criteria. Purposive sampling was adopted for the current research. It is because the availability of pediatric study samples was extremely low. Purposive sampling, recognized as judgmental, selective, or subjective sampling, is a pattern of non-probability sampling in that investigators trust their self-acumen while picking out subjects of the inhabitants to participate in their research.

Inclusion and Exclusion Criteria

Inclusion criteria: Patients with superficial partial thickness burn; burn covering 5-20% of TBSA; written informed consent was obtained from their parents. 

Exclusion criteria: Patients with burns involving the face, hands, feet, genitalia, perineum, and major joints with burn-wound infection; delayed arrival to hospital (more than 24 hours); patients with known allergies to honey or Ag-SD; patients with other systemic illnesses, e.g., protein energy malnutrition (PEM) and cerebral palsy.

Procedure of Data Collection, Data Analysis, and Interpretation 

The TBSA entangled in burn calculated through rules of nine [142-144]. Total samples were divided into two groups. Dressings for patients in Group-1 and Group-2 were conducted with pure, undiluted, and unprocessed honey and 1% Ag-SD, respectively. The honey utilized was of multi-floral origin. It was obtained from home garden of the principal researcher. Grouping was determined through a lottery among parents. General management was the same in both groups. Study variables were recorded on the third, fifth, seventh, tenth, and fourteenth day of treatment. Patients of both groups followed the same discharge criteria, and weekly follow-ups were done for up to four weeks. Infection rate, pathogenic microbes involved, and details were detailed in the result section. 

Data were collected, compiled, and statistical analysis was conducted by SPSS software version 20 [IBM Corp. Released 2011. IBM SPSS Statistics for Windows, Version 20.0. Armonk, NY: IBM Corp.]. The findings of the study were presented by frequency and percentage in tables. Means and standard deviations for continuous variables and frequency distributions for categorical variables were used to describe the characteristics of the total sample. To estimate the p-value, the non-parametric Mann-Whitney U test was used for non-normalize data, an independent sample t-test was used for normalized data, and a Chi-square test was applied for dichotomous variables. A p-value of <0.05 was considered significant.

Ethical Approval

This study obtained ethical approval from the Institutional Review Board (IRB) of the Dhaka Shishu Hospital, Dhaka-1000, Bangladesh (Reference No. BICH-ERC-9/2/2017, June 19, 2017). The study participants' parents (as participants were minors) were Informed in detail regarding the study plan and future publication. Only those participants who gave written informed consent from a valid guardian were included in this research.

Statistical Analysis Plan

Demographic data were furnished as mean±SD or median with interquartile range (IQR) or number with percent in parenthesis. To assess the association between demographic features and treatment group, a non-parametric Mann-Whitney U test for non-normalize data, an independent sample t-test for normalized data, and a Chi-square test was applied for dichotomous observation. The logistic regression model was used to estimate the treatment effects on the slough's presence and the slough's reduction on Days 3, 5, and 7. We also used a multivariate regression model to evaluate the mean difference in the completion of epithelialization between the treatment group. All the regression model was adjusted by age, sex, weight, cause of burn (hot water and cooking-related burn), duration of burn injury, and surface area. For statistical analysis, we used STAT-15, and a graphical presentation was performed by Graph Pad Prism 8.3. All the statistical significance was considered with p < 0.05. 

## Results

**Figure 1:**
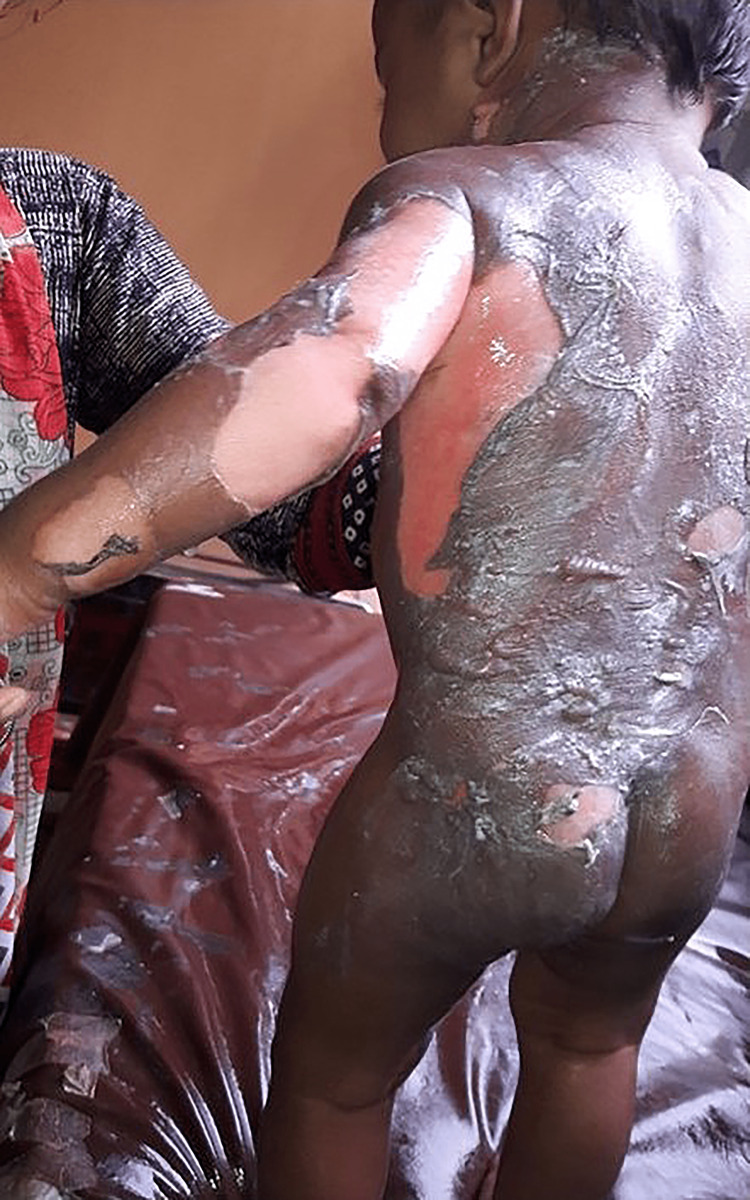
Photo depicting a patient in Group-1 on admission The photograph has been taken by Md. Moniruzzaman.

**Figure 2:**
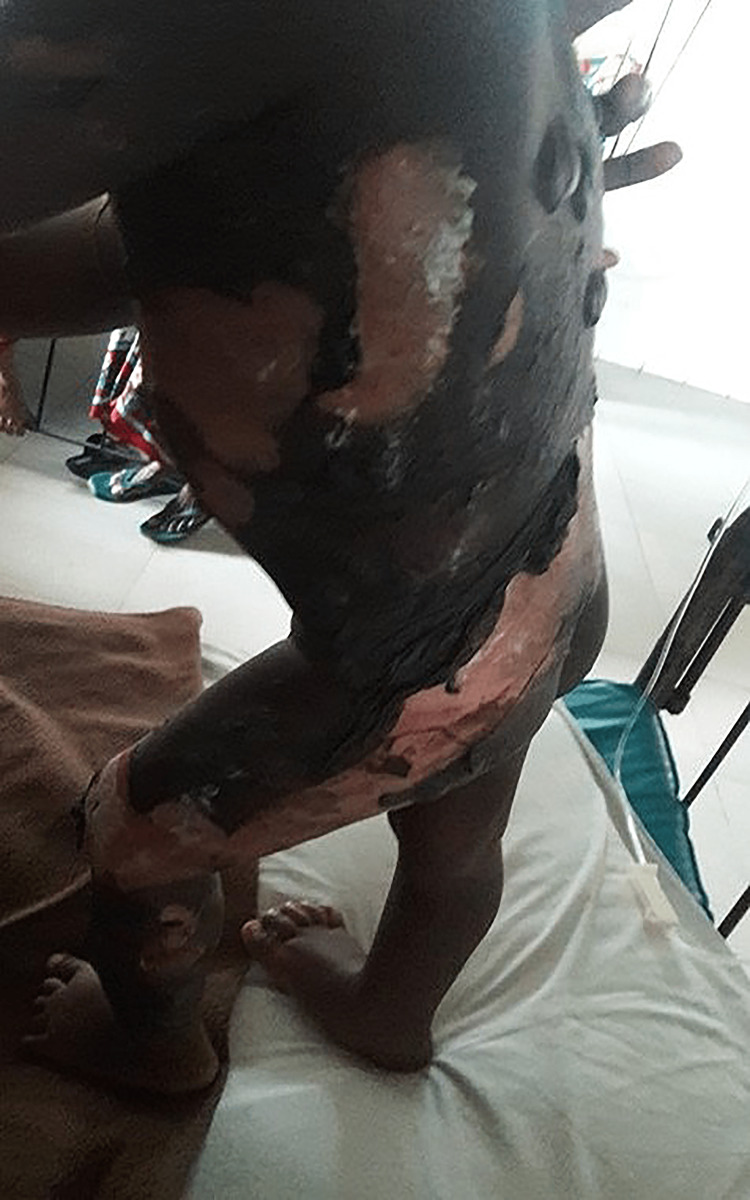
Photo depicting a patient in Group-2 on admission The photograph has been taken by Md. Moniruzzaman.

**Figure 3:**
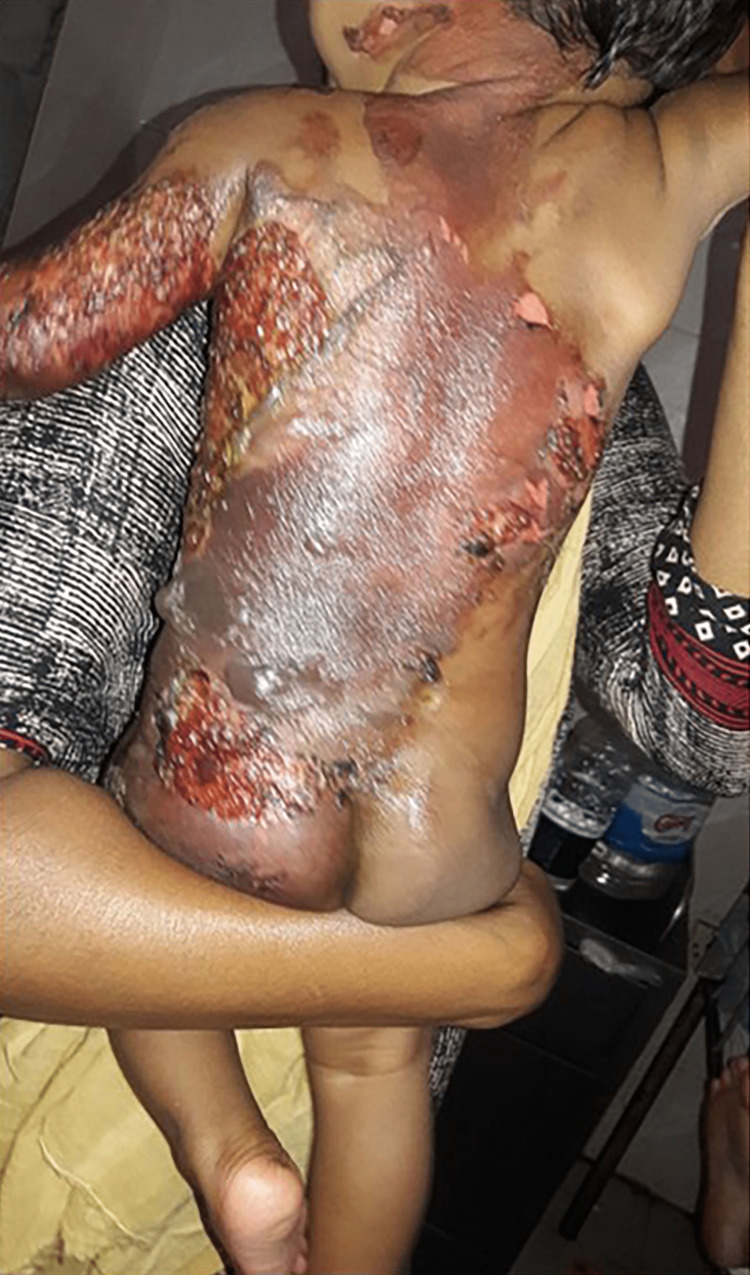
Photo depicting the patient in Group-1 on Day 3 of treatment The photograph has been taken by Md. Moniruzzaman.

**Figure 4:**
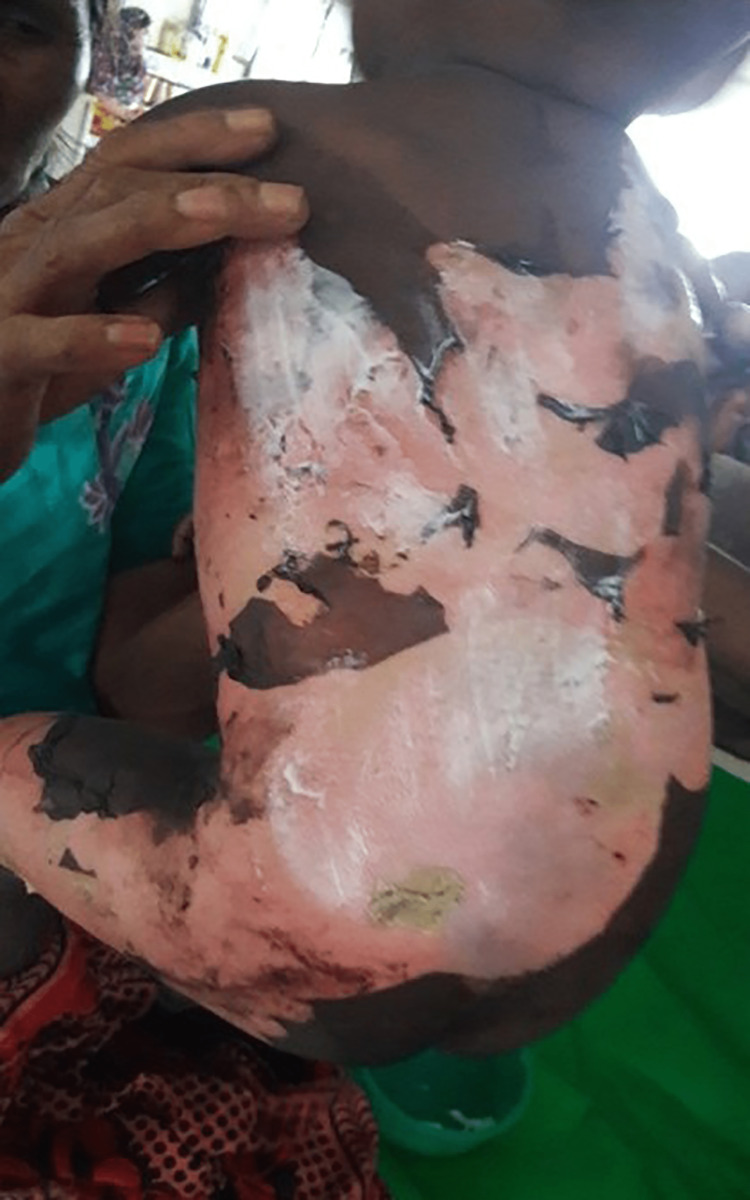
Photo depicting the patient in Group-2 on Day 3 of treatment The photograph has been taken byMd. Moniruzzaman.

**Figure 5:**
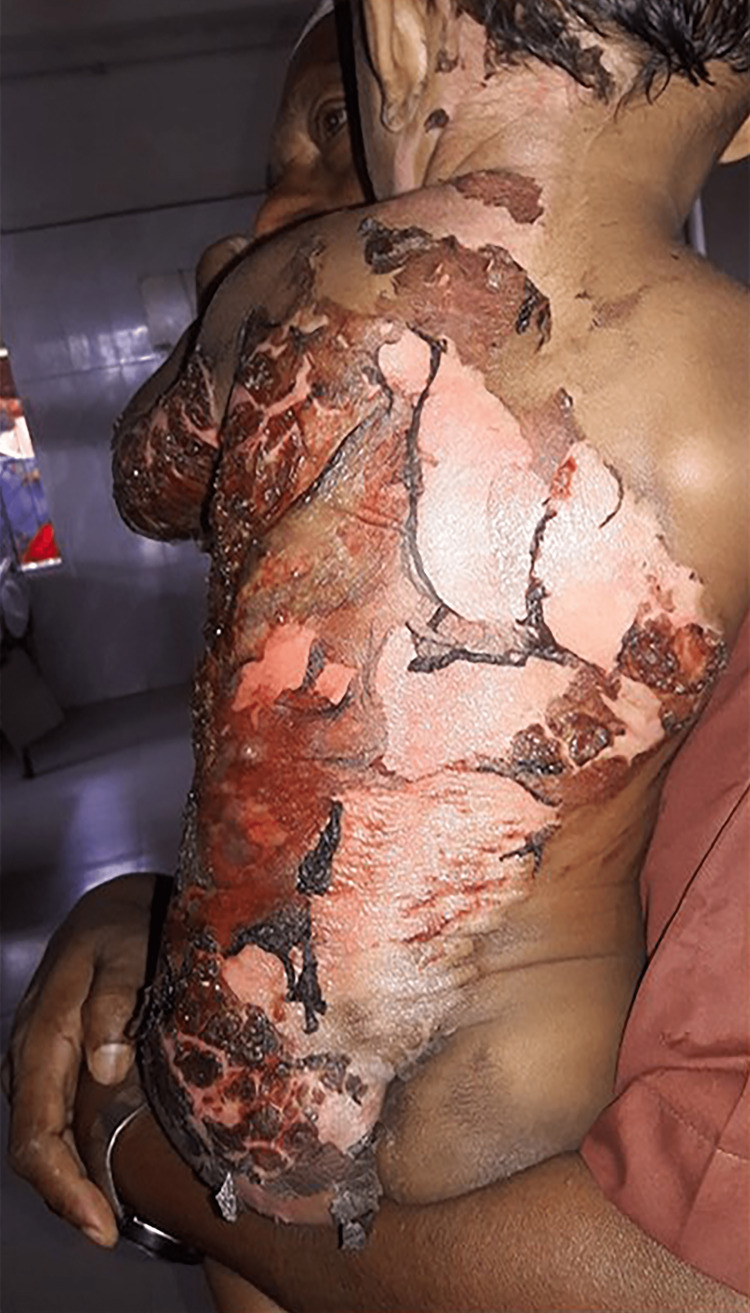
Photo depicting the patient in Group-1 on Day 7 of treatment The photograph has been taken by Md. Moniruzzaman.

**Figure 6:**
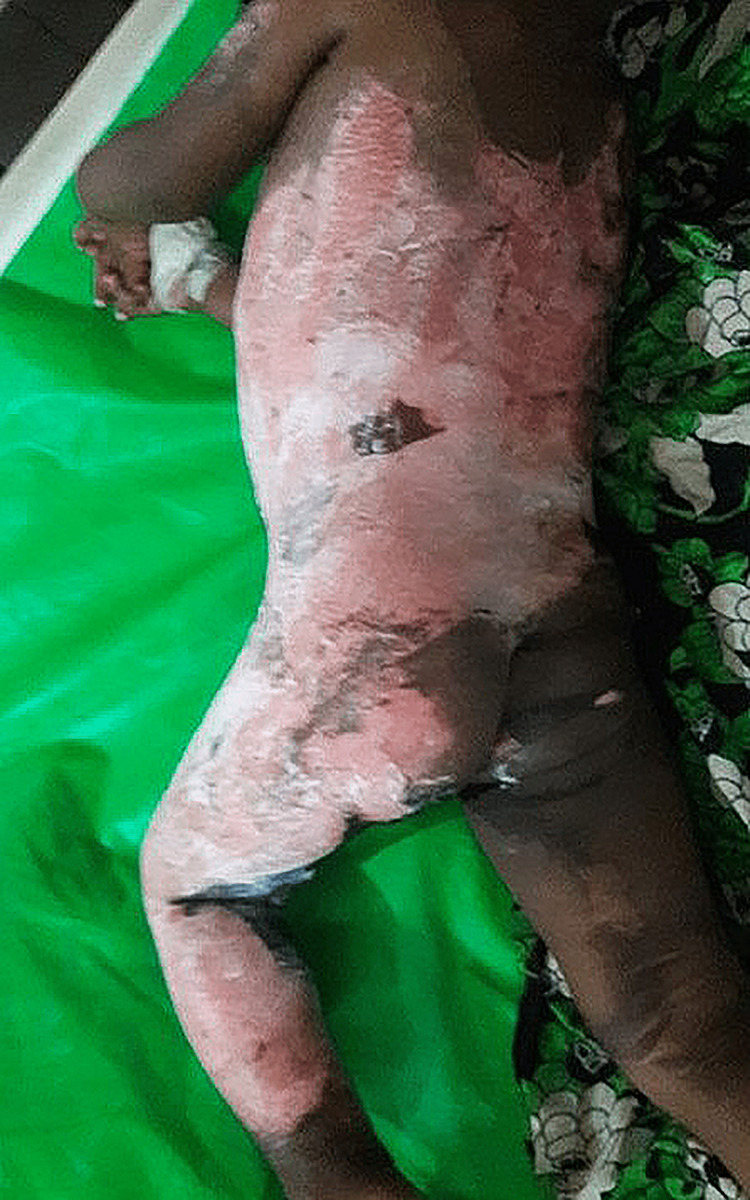
Photo depicting the patient in Group-2 on Day 7 of treatment The photograph has been taken by Md. Moniruzzaman.

**Figure 7:**
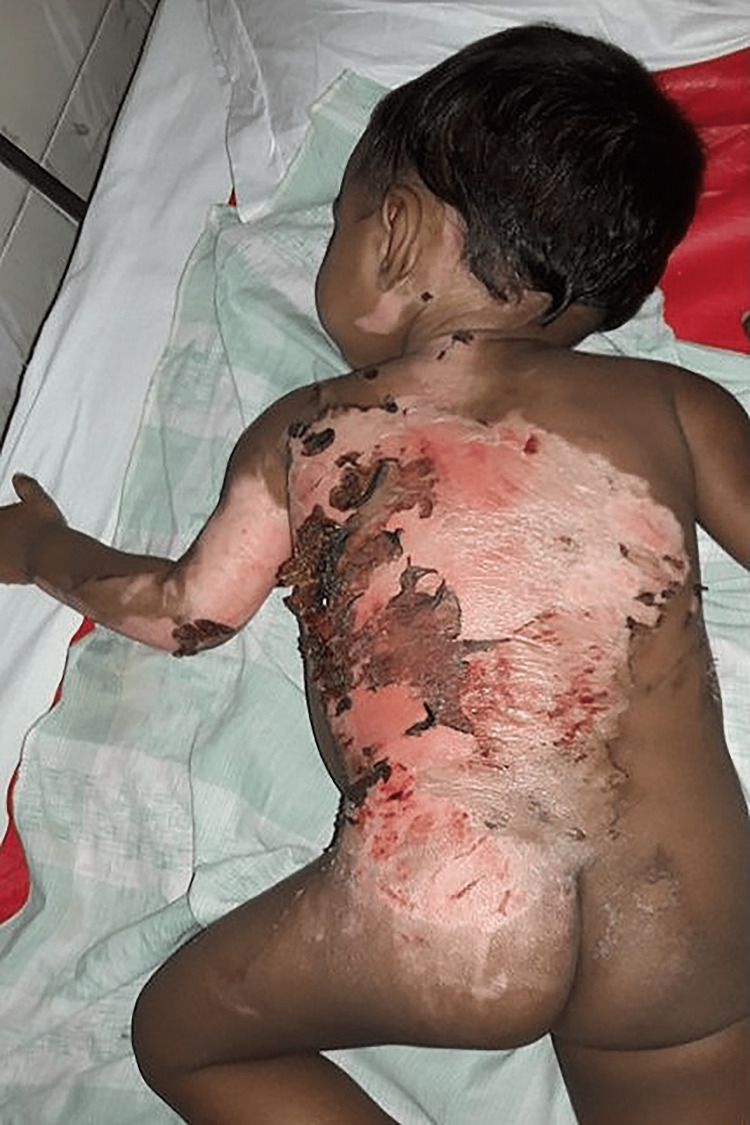
Photo depicting the patient in Group-1 on Day 10 of treatment The photograph has been taken by Md. Moniruzzaman.

**Figure 8:**
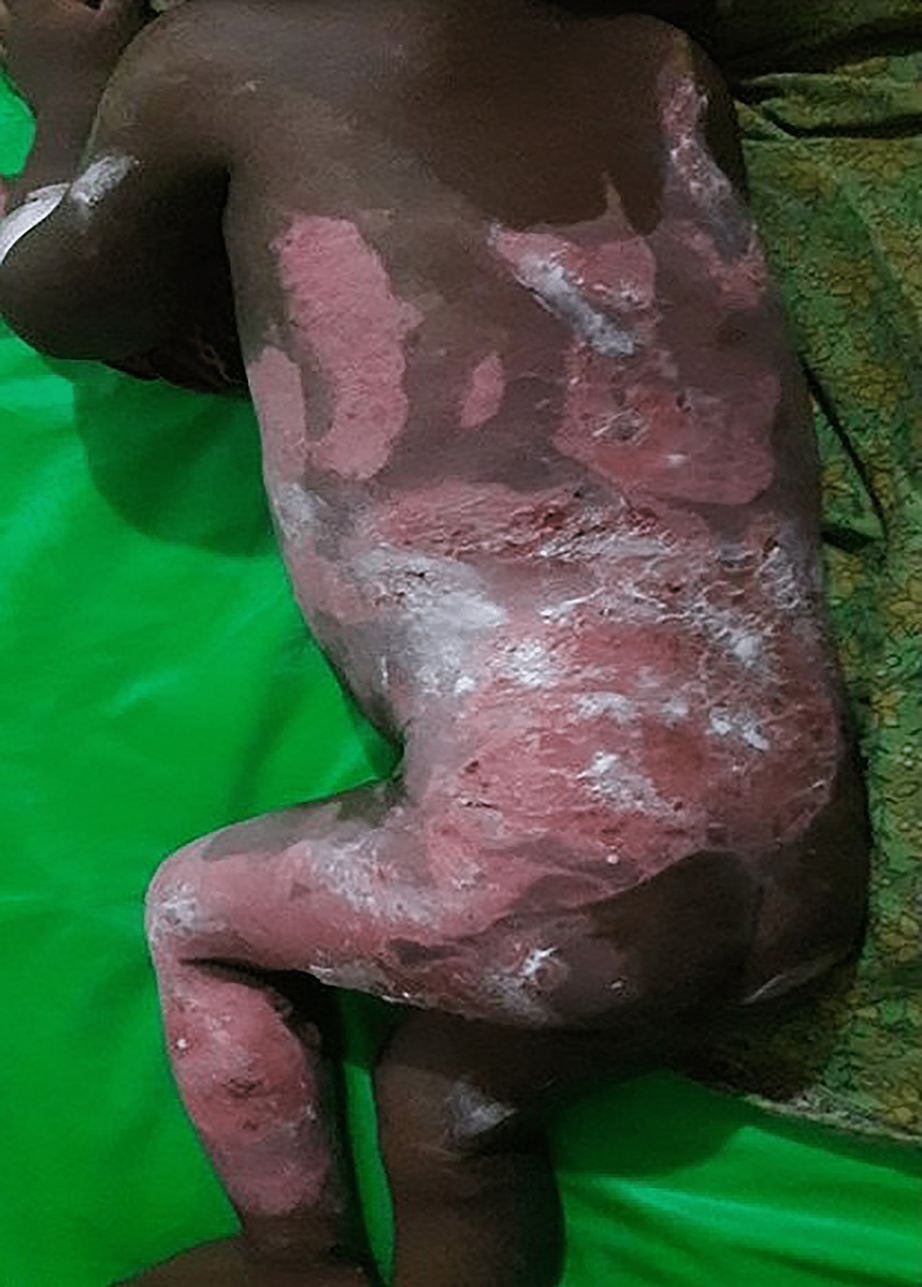
Photo depicting the patient in Group-2 on Day 10 of treatment The photograph has been taken by Md. Moniruzzaman.

In total, 70 participants, 35 in Group-1 and another 35 in Group-2, were enrolled to see the effectiveness of pediatric first-degree burn management. Burn patients' photographs are illustrated in Figures 1 and 2 on the admission of Groups I and II, respectively. The median age with the IQR was 24 (14, 40) in Group-1 and 19 (12, 60) months in Group-2. In Group-2, 21 (60%) were girls, whereas in Group-2, 15 (42.9%) were girls. Most of the study population (85.71%) were below 5 years. The median body weight of the studied participants was 10 kg in both groups. None of the observations showed any statistical significance (p > 0.05) in the baseline (Table 1). Figures 3 and 4 illustrate the treatment progress on Day 3 of Group-1 and Group-2, respectively. Similarly, Figures 5 and 6 pictorially denote the treatment progress on Day 7 among Group-1 and Group-2, respectively. Figures 7 and 8 display treatment progress on Day 10 in Group-1 and Group-2, respectively. 

**Table 1:** Socio-demographic features of the study participants Data were presented as median with IQR or number with percent in parenthesis. To estimate the p-value, the non-parametric Mann-Whitney U test was used for non-normalized data, an independent sample t-test was used for normalized data, and a Chi-square test was applied for dichotomous variables. IQR: Interquartile range

	Group-1 (n=35)	Group-2 (n=35)	p-value
Age in months, median (IQR)	24 (14, 40)	19 (12, 60)	0.972
Sex			
Boys	14 (40.0%)	20 (57.1%)	0.151
Girls	21 (60.0%)	15 (42.9%)	
Weight in kg, median (IQR)	10.0 (8.70, 14.0)	10.0 (9.0, 15.5)	0.292
Cause of burn			
Hot water	21 (60.0%)	23 (65.7%)	0.621
Cooking related burn	14 (40.0%)	12 (34.3%)	
Duration of burn injury	3.66±1.85	3.66±1.85	0.999
Total surface area of burns (%)	10.3±2.65	11.2±2.87	0.158

Infection details

In Group-1 and Group-2, 14.3% and 22.9% of participants developed wound infections. Pearson’s Chi-square test found no statistical differences between the two groups regarding wound infection (p > 0.05). In Group-1, most (60%) of the wound infection was caused by *Staphylococcus aureus*. Other organisms were *Escherichia coli* (20.0%) and *Pseudomonas aeruginosa ​*(20.0%). In Group-2, half (50.0%) of the wound infection was caused by *S. aureus*. Other organisms were *E. coli* (12.5%) and *P. aeruginosa* (37.5%).

**Figure 9:**
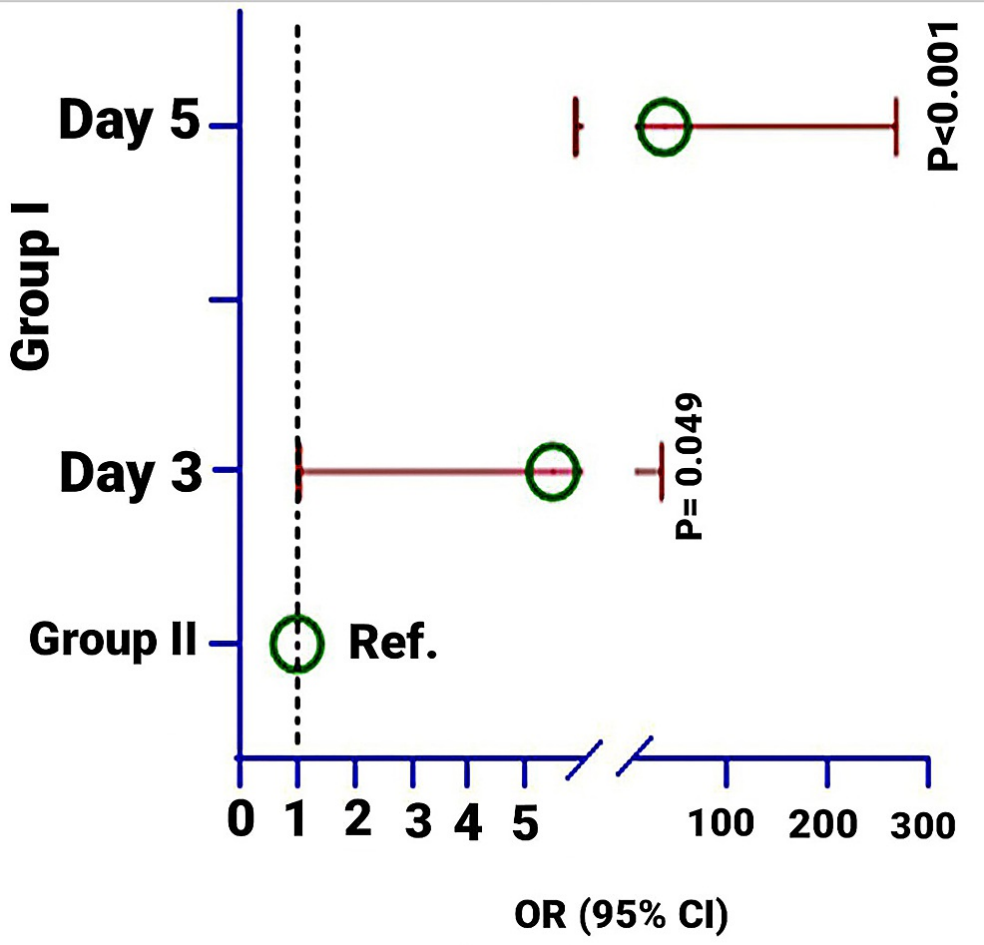
Odds of reduction of the slough at Days 3, 5, and 7 in Group-1 compared to Group-2. The multivariate logistic regression model was used to estimate the p-value. The model was adjusted by age, sex, weight, cause of burn (hot water and cooking-related burn), duration of burn injury, and surface area. The figure has been created by Md. Ahsanul Haq.

Multivariate logistic regression found that the odds ratio (OR) of the reduction of slough in Group-1 was 5.46 times (95% CI: 1.05, 37.9; p = 0.040) and 39.8 times (95% CI: 5.88, 269.5) higher at Days 3 and 5 compared to Group-2. This study observed that both groups were 100% slough was abolished at Day 7 (see Table 2 and Figure 9). 

**Table 2:** Association of treatment effects on the presence of slough at Days 3, 5, and 7 ^1 ^Non-parametric Chi-square test was used to estimate the p-value; ^2 ^Multivariate logistic regression model was used to calculate the p-value. The model was adjusted by age, sex, weight, cause of burn (hot water and cooking-related burn), duration of burn injury, and surface area. OR: Odds ratio

	Present	Absence	p-value^1^	OR (95% CI)	p-value^2^
Slough on day 3					
Group I	27 (77.1%)	8 (22.9%)	0.040	5.46 (1.05, 37.9)	0.049
Group II	33 (94.3%)	2 (5.71%)		Ref.	
Slough at day 5					
Group I	6 (17.1%)	29 (82.9%)	<0.001	39.8 (5.88, 269.5)	<0.001
Group II	27 (77.1%)	8 (22.9%)		Ref.	
Slough on day 7					
Group I	0	35 (100%)	-	-	-
Group II	0	35 (100%)		Ref.	

**Figure 10:**
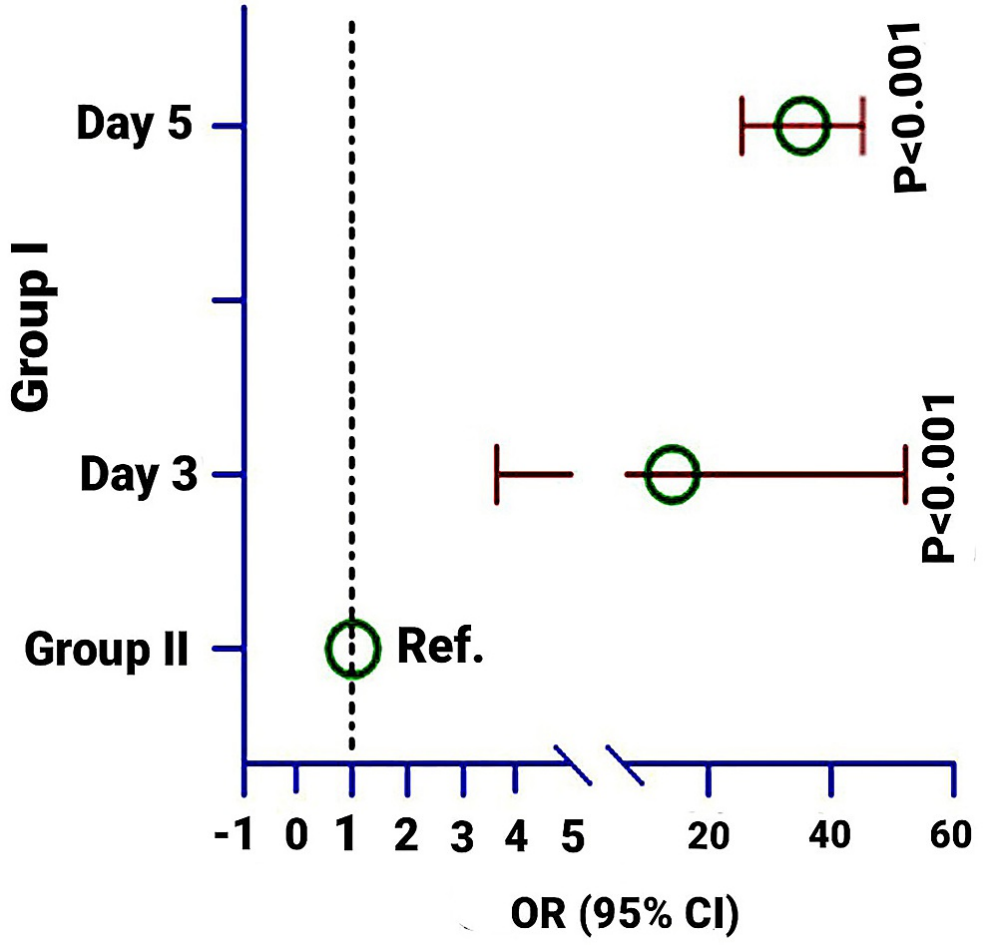
Odds of reduction of exudate at Days 3, 5, and 7 in Group-1 compared to Group-2. The multivariate regression model was used to estimate the p-value. The regression model was adjusted by age, sex, weight, cause of burn (hot water and cooking-related burn), duration of burn injury, and surface area. The figure has been created by Md. Ahsanul Haq.

The multivariate logistic regression model was used to estimate the p-value. The model was adjusted by age, sex, weight, cause of burn (hot water and cooking-related burn), duration of burn injury, and surface area. Reduction of exudate showed a significant OR in Group-1 compared to Group-2 on Days 3 (Figure 6) and 5 and which was 13.8 times (95% CI: 3.63, 52.2; p < 0.001) and 35.2 times (95% CI: 25.2, 45.2; p < 0.001) higher in Group-1 compared to Group-2. On Day 7, both groups reduced 100% exudate (see Table 3 and Figure 10).

**Table 3:** Association of treatment effects on the presence of exudate at Days 3, 5, and 7 in Group-1 compared to Group-2. ^1 ^Non-parametric Chi-square test was used to estimate the p-value; ^2 ^Multivariate logistic regression model was used to calculate the p-value. The model was adjusted by age, sex, weight, cause of burn (hot water and cooking-related burn), duration of burn injury, and surface area. OR: Odds ratio

	Present	Absence	p-value^1^	OR (95% CI)	p-value^2^
Exudate on Day 3					
Group-1	11(31.4%)	24 (68.6%)	<0.001	13.8 (3.63, 52.2)	<0.001
Group-2	29(82.9%)	6 (17.1%)		Ref.	
Exudate at Day 5					
Group-1	0	35 (100%)	<0.001	35.2 (25.2, 45.2)	<0.001
Group-2	21(60.0%)	14 (40.0%)		Ref.	
Exudate at Day 7					
Group-1	0	35 (100%)	-	-	-
Group-2	0	35 (100%)		Ref.	

Completion of epithelialization in Group-1 decreased by 11.3% compared to Group-2 (p < 0.001, 95% CI: -14.8, -7.79) on Day 7. Whereas completion of epithelialization in Group-1 increased by 9.29% (p = 0.006, 95% CI: 2.78, 15.8) on Day 10. An older person has a relatively lower completion of epithelialization by 0.15% (p = 0.027, 95% CI: -0.28, -0.02). A similar lower count was noted on Day 14 by 10% (p = 0.012, 95% CI: -0.17, -0.02) (Table 4).

**Table 4:** Mean difference of completion of epithelialization at Days 7, 10, and 14 in Group-1 compared to Group-2. The multivariate regression model was used to estimate the p-value. The regression model was adjusted by age, sex, weight, cause of burn (hot water and cooking-related burn), duration of burn injury, and surface area.

	Unadjusted		Adjusted	
	β-Coff (95% CI)	p-value	β-Coff (95% CI)	p-value
At day 7				
Group II	Ref.		Ref.	
Group I	-9.09 (-13.6, -4.58)	<0.001	-11.3 (-14.8, -7.79)	<0.001
At day 10				
Group II	Ref.		Ref.	
Group I	9.28 (2.91, 15.7)	0.005	9.29 (2.78, 15.8)	0.006
Age	-	-	-0.15 (-0.28, -0.02)	0.027
At day 14				
Group II	Ref.		Ref.	
Group I	2.71 (-0.91, 6.34)	0.140	2.57 (-1.13, 6.27)	0.170
Age			-0.10 (-0.17, -0.02)	0.012

Duration of hospitalization showed no significant difference and thus was not reported here. β-Coff (95% CI: -0.26(-1.38, 0.86), p = 0.642, but elderly age children left the hospital earlier by 0.04 days (β=0.04, 95% CI: 0.02, 0.06, p = 0.001). In the overall group, elderly children had a higher probability of leaving the hospital early.

## Discussion

Burns remain the fourth typical cause of injury among the pediatric population, often demanding multidisciplinary medical care [145-147]. It has been observed that increased morbidities and mortalities occur, principally among LMICs, because of burns [147-149]. Microbial infection, followed by sepsis, is among the highest contributing features in burn-related impermanence and fatal clinical outcome [150, 151]. Burn patients quickly develop fluid and electrolyte imbalances that often cause fatality. Proper and adequate fluid and electrolyte correction saves pediatric and adult lives [152-155].

This study population's age range was 6 to 120 months. Earlier, one Bangladeshi study reported that the highest rate of burn cases was among 1-4 years and 3.5-fold higher than among teenagers (15-17 years) [156]. Another study reported that the range of these burn cases was 0-14 years [157]. Our findings were in line with earlier studies [156, 158]. There were no statistically significant differences observed among pediatric burn cases regarding sex. Our results were similar to an earlier study conducted in the USA and Italy [158,159]. Nevertheless, one study at Linköping University Hospital of Sweden reported that more males (70%) were burned cases than females [160]. While conducting logistic regression analysis, one recent Malawian study reported no statistically significant difference regarding the fatal outcome in the odds when compared between sex (OR 1.12, 95% CI: 0.82-1.52, p = 0.5) [161]. The current study participants of childhood burn patients’ median weight was 10 kg. Most of our cases were burned through hot water and associated with cooking. Our findings were similar to an earlier study of the USA extracting data from the “Electronic Injury Surveillance System Database” from 2000-2016 [162]. Another study conducted in Ghana reported that most pediatric burns were caused by open-fire cooking utilizing wood, manure, garbage, etc. [163]. To reach the nearest hospital and the treatment process at home, pediatric burn cases at home require over three hours in Bangladesh. Two US studies revealed that the median time from commencing burn to transport to the burn care hospital was 6.26 (range: 0.5-96 hours) and 7.2 hours (range: 1.6-48) [164,165]. Nonetheless, one more study reported that from Central Malawi to Kamuzu Central Hospital was within eight hours [166]. It seems transportation in our study was lower than in earlier studies. It has been reported that a prolonged time to reach the hospital enhances morbidities and mortalities [167,168]. This study found that the TBSA (%) was 10.3 to 11.2. It has been reported that most pediatric burns are of accidental origin and often lead to death [11,35]. Less than 10% of TBSA are considered minor among childhood cases [169]. Nevertheless, a substantial proportion of pediatric burns involve over 15% TBSA, which remains as dominant cause of the commencement of the systemic inflammatory response syndrome (SIRS) [170]. SIRS usually shows unfocused symptoms and is often instigated by a pernicious stressor, such as trauma, inflammation, ischemia, infection, and/or various offending agents alone or in combination. Thereby showing an over-elaborated immune response [171].

This study revealed that childhood burn exudate and slough statistically significantly eliminated earlier among cases were treated with honey when compared with the 1% Ag-SD-treated group through multivariate logistic regression model analysis was applied. Multiple studies reported that honey dressings were more effective in sterilizing and fostering medicinal and curative approaches to manage superficial and partial thickness burn wounds than Ag-SD [138,172-174]. 

Study Group-2 (1% Ag-SD) shows a statistically significantly improved epithelization process than the honey-treated group by Day 7. Multiple studies reported that honey shows better efficacy regarding re-epithelialization [173,175,176]. The current study revealed that the reverse situation was observed on Day 10, as honey treated shows a much better epithelization process when compared with Ag-SD-treated group. Nevertheless, one Indonesian study revealed no statistically significant differences between the honey and Ag-SD-treated group regarding re-epithelization on Day 10 [177]. However, one Indian study revealed that the honey-treated group showed at least two weeks earlier healing than the Ag-SD group [178]. Baghel et al. reported that burn wound disinfects and heals faster when treated with honey than Ag-SD. Compared to 1% Ag-SD, honey stopped hypertrophic disfigurement and post-burn tightening and narrowing, and reduced the requirement of debridement regardless of hospitalization time [179]. The superficial and partial-thickness burns' re-epithelialization and the healing process were significant with high speed when burn-wound treated with honey than 1% Ag-SD (13·47±4·06 Vs. 15·62±4·40 days, respectively: p < 0.0001) [173]. Additionally, older childhood burn cases' re-epithelialization process was slower than relatively younger children in the current study. It has been reported that disregarding the cause of burn wounds; elderly victims manifest a slow-moving healing process with enhanced complications. With the aging process reduced thereby, the immune system promotes infections, the healing process is slowed down, and develops various complications [180-182]. Although, no statistically significant variance was observed between the honey and Ag-SD-treated group regarding hospital stay. However, another study reported that honey-treated cases took at least 3 days less than 1% Ag-SD to heal entirely and be released from the hospital [173]. One more study revealed that honey substantially reduced infection by day 5 and pain. Thereby minimizing hospital stay among burn cases [120,183].

Limitations of this research

This study sample size was small. If it were more extensive, the results would be more accurate. Researchers have time and financial constraints. These are significant obstacles to conducting this research more widely.

## Conclusions

The present study manifests better clinical outcomes regarding burn traumas dressed by honey than in Ag-SD. It increases the rate of healing. It also improves overall patient compliance and significantly reduces the cost of dressing which is a crucial factor in burn wound management. So, for good analgesic effect, a minimum rate of hypertrophic scar and post-burn contracture, less expensive and economically feasible for LMICs, and wide accessibility make honey an exemplary dressing agent for treating burns. Further studies regarding this issue involving large-scale study samples and multicenter research are advocated. Thereby, study results can be generalized all over the country. 

## References

[1] Jeschke MG, van Baar ME, Choudhry MA, Chung KK, Gibran NS, Logsetty S (2020). Burn injury. Nat Rev Dis Primers.

[2] Friedstat J, Brown DA, Levi B (2017). Chemical, electrical, and radiation Injuries. Clin Plast Surg.

[3] Nielson CB, Duethman NC, Howard JM, Moncure M, Wood JG (2017). Burns: pathophysiology of systemic complications and current management. J Burn Care Res.

[4] Moi AL, Haugsmyr E, Heisterkamp H (2016). Long-term study of health and quality of life after burn injury. Ann Burns Fire Disasters.

[5] Lateef Z, Stuart G, Jones N, Mercer A, Fleming S, Wise L (2019). The cutaneous inflammatory response to thermal burn injury in a murine model. Int J Mol Sci.

[6] Zhang P, Zou B, Liou YC, Huang C (2021). The pathogenesis and diagnosis of sepsis post burn injury. Burns Trauma.

[7] Ziolkowski N, Kitto SC, Jeong D, Zuccaro J, Adams-Webber T, Miroshnychenko A, Fish JS (2019). Psychosocial and quality of life impact of scars in the surgical, traumatic and burn populations: a scoping review protocol. BMJ Open.

[8] Simons M, Price N, Kimble R, Tyack Z (2016). Patient experiences of burn scars in adults and children and development of a health-related quality of life conceptual model: a qualitative study. Burns.

[9] Mathias E, Srinivas Murthy M (2017). Pediatric thermal burns and treatment: a review of progress and future prospects. Medicines (Basel).

[10] Ricciardello D, Lee M, Tran S, Chamberlain K, Holland AJ, Bertinetti M (2022). Laryngotracheal stenosis post mechanical ventilation in paediatric burns patients. Int J Burns Trauma.

[11] Moehrlen T, Szucs T, Landolt MA, Meuli M, Schiestl C, Moehrlen U (2018). Trauma mechanisms and injury patterns in pediatric burn patients. Burns.

[12] Biasini A, Biasini M, Stella M (2014). Intensive care of children with burn injuries and the role of the multidisciplinary team. Nurs Child Young People.

[13] Karam E, Lévesque MC, Jacquemin G (2014). Building a multidisciplinary team for burn treatment - lessons learned from the Montreal tendon transfer experience. Ann Burns Fire Disasters.

[14] Williams FN, Herndon DN, Hawkins HK (2009). The leading causes of death after burn injury in a single pediatric burn center. Crit Care.

[15] Dokter J, Felix M, Krijnen P, Vloemans JF, Baar ME, Tuinebreijer WE, Breederveld RS (2015). Mortality and causes of death of Dutch burn patients during the period 2006-2011. Burns.

[16] Guillory AN, Clayton RP, Herndon DN, Finnerty CC (2016). Cardiovascular dysfunction following burn injury: what we have learned from rat and mouse models. Int J Mol Sci.

[17] Salehi SH, As'adi K, Naderan M, Shoar S, Saberi M (2016). Assessment of erectile dysfunction following burn injury. Urology.

[18] Gallo RL (2017). Human skin is the largest epithelial surface for interaction with microbes. J Invest Dermatol.

[19] Kim JY, Dao H (2022 ). Physiology, integument. https://www.ncbi.nlm.nih.gov/books/NBK554386/.

[20] Institute for Quality and Efficiency in Health Care (2019). How does skin work?. https://www.ncbi.nlm.nih.gov/books/NBK279255/.

[21] Ibrahim AAE, Bagheran N, Smoller B, Reyes-Baron C, Bagherani N. (2021). Functions of the skin. Atlas of Dermatology, Dermatopathology, and Venereology.

[22] Schaefer TJ, Szymanski KD (2022). Burn evaluation and management. https://www.ncbi.nlm.nih.gov/books/NBK430741/.

[23] Noorbakhsh SI, Bonar EM, Polinski R, Amin MS (2021). Educational case: burn injury - pathophysiology, classification, and treatment. Acad Pathol.

[24] Hege AR, Choubisa CA, Kasatwar P (2022). Physiotherapeutic rehabilitation of a patient following an electrical burn: a case report. Cureus.

[25] Markiewicz-Gospodarek A, Kozioł M, Tobiasz M, Baj J, Radzikowska-Büchner E, Przekora A (2022). Burn wound healing: clinical complications, medical care, treatment, and dressing types: the current state of knowledge for clinical practice. Int J Environ Res Public Health.

[26] Warby R, Maani CV (2022). Burn classification. https://www.ncbi.nlm.nih.gov/books/NBK539773/.

[27] Pencle FJ, Mowery ML, Zulfiqar H (2022). First degree burn. https://www.ncbi.nlm.nih.gov/books/NBK442021/.

[28] AlAlwan MA, Almomin HA, Shringarpure SD (2022). Survival from ninety-five percent total body surface area burn: a case report and literature review. Cureus.

[29] Datta PK, Roy Chowdhury S, Aravindan A (2022). Medical and surgical care of critical burn patients: a comprehensive review of current evidence and practice. Cureus.

[30] James SL, Lucchesi LR, Bisignano C (2020). Epidemiology of injuries from fire, heat and hot substances: global, regional and national morbidity and mortality estimates from the Global Burden of Disease 2017 study. Inj Prev.

[31] He S, Alonge O, Agrawal P, Sharmin S, Islam I, Mashreky SR, Arifeen SE (2017). Epidemiology of burns in rural Bangladesh: an update. Int J Environ Res Public Health.

[32] Mashreky SR, Rahman A, Svanström L, Khan TF, Rahman F (2011). Burn mortality in Bangladesh: findings of national health and injury survey. Injury.

[33] Price K, Lee KC, Woolley KE, Falk H, Peck M, Lilford R, Moiemen N (2021). Burn injury prevention in low- and middle- income countries: scoping systematic review. Burns Trauma.

[34] Tusiime M, Musoke D, Muneza F, Mutto M, Kobusingye O (2022). Prevalence, risk factors and perceptions of caregivers on burns among children under 5 years in Kisenyi slum, Kampala, Uganda. Inj Epidemiol.

[35] Toon MH, Maybauer DM, Arceneaux LL, Fraser JF, Meyer W, Runge A, Maybauer MO (2011). Children with burn injuries--assessment of trauma, neglect, violence and abuse. J Inj Violence Res.

[36] Carlsson A, Udén G, Håkansson A, Karlsson ED (2006). Burn injuries in small children, a population-based study in Sweden. J Clin Nurs.

[37] Oseni OG, Olamoyegun KD, Olaitan PB (2017). Paediatric burn epidemiology as a basis for developing a burn prevention program. Ann Burns Fire Disasters.

[38] Alharthy N, Al Mutairi M, AlQueflie S, Nefesa AB, Manie NB, Nafesa SB, Al Zahrani FS (2016). Pattern of burns identified in the pediatrics emergency department at King Abdul-Aziz Medical City: Riyadh. J Nat Sci Biol Med.

[39] Padilla PL, Freudenburg EP, Kania K, Laney RW, Branski LK, Herndon DN (2018). Negative pressure wound therapy with instillation and dwell for the management of a complex burn: a case report and review of the literature. Cureus.

[40] Bittner EA, Shank E, Woodson L, Martyn JA (2015). Acute and perioperative care of the burn-injured patient. Anesthesiology.

[41] Stokes MA, Johnson WD (2017). Burns in the Third World: an unmet need. Ann Burns Fire Disasters.

[42] Gupta JL, Makhija LK, Bajaj SP (2010). National programme for prevention of burn injuries. Indian J Plast Surg.

[43] Huff ML, Blome-Eberwein S (2022). Providencia rettgeri infection compromising post-burn recovery: a lesson in the importance of follow-up care. Cureus.

[44] Church D, Elsayed S, Reid O, Winston B, Lindsay R (2006). Burn wound infections. Clin Microbiol Rev.

[45] Vinaik R, Barayan D, Shahrokhi S, Jeschke MG (2019). Management and prevention of drug resistant infections in burn patients. Expert Rev Anti Infect Ther.

[46] Greenhalgh DG, Saffle JR, Holmes JH 4th (2007). American Burn Association consensus conference to define sepsis and infection in burns. J Burn Care Res.

[47] Pompermaier L, Steinvall I, Elmasry M, Thorfinn J, Sjöberg F (2018). Burned patients who die from causes other than the burn affect the model used to predict mortality: a national exploratory study. Burns.

[48] Norbury W, Herndon DN, Tanksley J, Jeschke MG, Finnerty CC (2016). Infection in burns. Surg Infect (Larchmt).

[49] Norman G, Christie J, Liu Z (2017). Antiseptics for burns. Cochrane Database Syst Rev.

[50] Tran S, Jacques MA, Holland AJ (2019). Assessment and management of minor burns in children. Aust J Gen Pract.

[51] Cox SG, Martinez R, Glick A, Numanoglu A, Rode H (2015). A review of community management of paediatric burns. Burns.

[52] Hyland EJ, Connolly SM, Fox JA, Harvey JG (2015). Minor burn management: potions and lotions. Aust Prescr.

[53] The Royal Children's Hospital Melbourne (2022). Burns - acute management. https://www.rch.org.au/clinicalguide/guideline_index/Burns/ .

[54] Maghsoudi H, Aghamohammadzadeh N, Khalili N (2008). Burns in diabetic patients. Int J Diabetes Dev Ctries.

[55] Chang CK, Bartkova J, Liao YS, Tzeng YS (2021). Successful treatment of lower limb burn wounds with long-term survived human skin allograft in an immunosuppressed patient: a case report. Int J Low Extrem Wounds.

[56] Marwa NP, Tarimo EA (2019). Provision of care to hospitalized pediatric burn patients: a qualitative study among nurses at Muhimbili National Hospital, Dar es Salaam, Tanzania. BMC Nurs.

[57] Jeschke MG, Peck MD (2017). Burn care of the elderly. J Burn Care Res.

[58] Abu-Sittah GS, Chahine FM, Janom H (2016). Management of burns in the elderly. Ann Burns Fire Disasters.

[59] Sevgi M, Toklu A, Vecchio D, Hamblin MR (2013). Topical antimicrobials for burn infections - an update. Recent Pat Antiinfect Drug Discov.

[60] Nethery W, Warner P, Durkee P, Dwyer A, Zembrodt J, Fowler L (2020). Efficacy of topical antimicrobial agents against bacterial isolates from burn wounds. J Burn Care Res.

[61] Cancio LC (2021). Topical antimicrobial agents for burn wound care: history and current status. Surg Infect (Larchmt).

[62] Dai T, Huang YY, Sharma SK, Hashmi JT, Kurup DB, Hamblin MR (2010). Topical antimicrobials for burn wound infections. Recent Pat Antiinfect Drug Discov.

[63] Oaks RJ, Cindass R (2022). Silver sulfadiazine. https://www.ncbi.nlm.nih.gov/books/NBK556054/.

[64] Brown M, Dalziel SR, Herd E, Johnson K, Wong She R, Shepherd M (2016). A randomized controlled study of silver-based burns dressing in a pediatric emergency department. J Burn Care Res.

[65] Verbelen J, Hoeksema H, Heyneman A, Pirayesh A, Monstrey S (2014). Aquacel(®) Ag dressing versus Acticoat™ dressing in partial thickness burns: a prospective, randomized, controlled study in 100 patients. Part 1: burn wound healing. Burns.

[66] Cooper RA (2007). Iodine revisited. Int Wound J.

[67] Gupta S, Shinde RK, Shinde S (2022). Comparison of the outcomes of cadexomer iodine and povidone-iodine ointments in wound management. Cureus.

[68] Shrestha A, Duwadi D, Jukosky J, Fiering SN (2019). Cecropin-like antimicrobial peptide protects mice from lethal E.coli infection. PLoS One.

[69] Palmieri TL, Greenhalgh DG (2002). Topical treatment of pediatric patients with burns: a practical guide. Am J Clin Dermatol.

[70] Modak SM, Fox CL Jr (1981). Sulfadiazine silver-resistant pseudomonas in burns: new topical agents. Arch Surg.

[71] Mabvuure NT, Brewer CF, Gervin K, Duffy S (2020). The use of moist exposed burn ointment (MEBO) for the treatment of burn wounds: a systematic review. J Plast Surg Hand Surg.

[72] Yong HY, Koh MS, Moon A (2009). The p38 MAPK inhibitors for the treatment of inflammatory diseases and cancer. Expert Opin Investig Drugs.

[73] Lee JK, Kim NJ (2017). Recent advances in the inhibition of p38 MAPK as a potential strategy for the treatment of Alzheimer's disease. Molecules.

[74] Carter D, Warsen A, Mandell K, Cuschieri J, Maier RV, Arbabi S (2014). Delayed topical p38 MAPK inhibition attenuates full-thickness burn wound inflammatory signaling. J Burn Care Res.

[75] Hamouda T, Hayes MM, Cao Z (1999). A novel surfactant nanoemulsion with broad-spectrum sporicidal activity against Bacillus species. J Infect Dis.

[76] Bahramsoltani R, Farzaei MH, Rahimi R (2014). Medicinal plants and their natural components as future drugs for the treatment of burn wounds: an integrative review. Arch Dermatol Res.

[77] Regan A, Hotwagner DT (2022). Burn fluid management. https://www.ncbi.nlm.nih.gov/books/NBK534227/.

[78] Moreira E, Burghi G, Manzanares W (2018). Update on metabolism and nutrition therapy in critically ill burn patients. Med Intensiva (Engl Ed).

[79] Chaudhary NA, Munawar MD, Khan MT (2019). Epidemiology, bacteriological profile, and antibiotic sensitivity pattern of burn wounds in the burn unit of a tertiary care hospital. Cureus.

[80] Tiwari VK (2012). Burn wound: how it differs from other wounds?. Indian J Plast Surg.

[81] Goel A, Shrivastava P (2010). Post-burn scars and scar contractures. Indian J Plast Surg.

[82] Challita R, Bazzi N, Fazaa E (2022). Management of burn scars: a five-year retrospective study. Cureus.

[83] Chang LS, Kim YH, Kim SW (2021). Reconstruction of burn scar contracture deformity of the extremities using thin thoracodorsal artery perforator free flaps. ANZ J Surg.

[84] Griggs C, Goverman J, Bittner EA, Levi B (2017). Sedation and pain management in burn patients. Clin Plast Surg.

[85] Gamst-Jensen H, Vedel PN, Lindberg-Larsen VO, Egerod I (2014). Acute pain management in burn patients: appraisal and thematic analysis of four clinical guidelines. Burns.

[86] Pardesi O, Fuzaylov G (2017). Pain management in pediatric burn patients: review of recent literature and future directions. J Burn Care Res.

[87] Lee KC, Joory K, Moiemen NS (2014). History of burns: the past, present and the future. Burns Trauma.

[88] Ullah MS, Noor-Ul Ferdous KM, Haider MM, Sarwar MKA, Alam MR, Khan A (2013). Management of paediatric second-degree burn in a developing country: with or without closed dressing?. Chattagram Maa-O-Shishu Hosp Med Coll J.

[89] Hudspith J, Rayatt S (2004). First aid and treatment of minor burns. BMJ.

[90] Kalanzi EW, O'Hara LM, O'Hara NN, Boyle JC (2014). Bed net related burns at Mulago national referral hospital, Uganda: a case series report. Burns.

[91] Sunny B, Sulthana L, James A, Sivakumar T (2016). Maggot infestation: various treatment modalities. J Am Coll Clin Wound Spec.

[92] McIntosh MD, Merritt RW, Kolar RE, Kimbirauskas RK (2011). Effectiveness of wound cleansing treatments on maggot (Diptera, Calliphoridae) mortality. Forensic Sci Int.

[93] Olawoye OA, Osinupebi OO, Ayoade BA (2013). Open burn wound dressing: a practical option in resource constrained settings. Ann Burns Fire Disasters.

[94] Mofazzal Jahromi MA, Sahandi Zangabad P, Moosavi Basri SM (2018). Nanomedicine and advanced technologies for burns: preventing infection and facilitating wound healing. Adv Drug Deliv Rev.

[95] Shpichka A, Butnaru D, Bezrukov EA (2019). Skin tissue regeneration for burn injury. Stem Cell Res Ther.

[96] Kaddoura I, Abu-Sittah G, Ibrahim A, Karamanoukian R, Papazian N (2017). Burn injury: review of pathophysiology and therapeutic modalities in major burns. Ann Burns Fire Disasters.

[97] Ranjbar R, Owlia P, Saderi H (2011). Characterization of Pseudomonas aeruginosa strains isolated from burned patients hospitalized in a major burn center in Tehran, Iran. Acta Med Iran.

[98] Yabanoglu H, Basaran O, Aydogan C, Azap OK, Karakayali F, Moray G (2013). Assessment of the effectiveness of silver-coated dressing, chlorhexidine acetate (0.5%), citric acid (3%), and silver sulfadiazine (1%) for topical antibacterial effects against the multi-drug resistant Pseudomonas aeruginosa infecting full-skin thickness burn wounds on rats. Int Surg.

[99] Tsolakidis S, Freytag DL, Dovern E (2022). Infections in burn patients: a retrospective view over seven years. Medicina (Kaunas).

[100] Lachiewicz AM, Hauck CG, Weber DJ, Cairns BA, van Duin D (2017). Bacterial infections after burn injuries: impact of multidrug resistance. Clin Infect Dis.

[101] Thombs BD, Bresnick MG (2022). Mortality risk and length of stay associated with self-inflicted burn injury: evidence from a national sample of 30,382 adult patients. Crit Care Med.

[102] Cambiaso-Daniel J, Boukovalas S, Bitz GH, Branski LK, Herndon DN, Culnan DM (2018). Topical antimicrobials in burn care: part 1-topical antiseptics. Ann Plast Surg.

[103] Khan S, John JR, Sharma RK (2021). Burn wounds, silver sulphadiazine and interpretations of serum Ag levels. Burns.

[104] Atiyeh BS, Costagliola M, Hayek SN, Dibo SA (2007). Effect of silver on burn wound infection control and healing: review of the literature. Burns.

[105] Levin NJ, Erben Y, Li Y, Brigham TJ, Bruce AJ (2022). A systematic review and meta-analysis comparing burn healing outcomes between silver sulfadiazine and aloe vera. Cureus.

[106] Shahzad MN, Ahmed N (2013). Effectiveness of aloe vera gel compared with 1% silver sulphadiazine cream as burn wound dressing in second-degree burns. J Pak Med Assoc.

[107] Hoffmann S (1984). Silver sulfadiazine: an antibacterial agent for topical use in burns. Scand J Plast Reconstr Surg.

[108] Fuller FW (2009). The side effects of silver sulfadiazine. J Burn Care Res.

[109] Razavi H, Darvishi MH, Janfaza S (2018). Silver sulfadiazine encapsulated in lipid-based nanocarriers for burn treatment. J Burn Care Res.

[110] Nímia HH, Carvalho VF, Isaac C, Souza FÁ, Gemperli R, Paggiaro AO (2019). Comparative study of silver sulfadiazine with other materials for healing and infection prevention in burns: a systematic review and meta-analysis. Burns.

[111] Maghsoudi H, Monshizadeh S, Mesgari M (2011). A comparative study of the burn wound healing properties of saline-soaked dressing and silver sulfadiazine in rats. Indian J Surg.

[112] Adhya A, Bain J, Ray O (2014). Healing of burn wounds by topical treatment: a randomized controlled comparison between silver sulfadiazine and nano-crystalline silver. J Basic Clin Pharm.

[113] Rosenkranz HS, Coward JE, Wlodkowski TJ, Carr HS (1974). Properties of silver sulfadiazine-resistant Enterobacter cloacae. Antimicrob Agents Chemother.

[114] Subrahmanyam M (2007). Topical application of honey for burn wound treatment - an overview. Ann Burns Fire Disasters.

[115] Eteraf-Oskouei T, Najafi M (2013). Traditional and modern uses of natural honey in human diseases: a review. Iran J Basic Med Sci.

[116] Yupanqui Mieles J, Vyas C, Aslan E, Humphreys G, Diver C, Bartolo P (2022). Honey: an advanced antimicrobial and wound healing biomaterial for tissue engineering applications. Pharmaceutics.

[117] Meo SA, Al-Asiri SA, Mahesar AL, Ansari MJ (2017). Role of honey in modern medicine. Saudi J Biol Sci.

[118] Zumla A, Lulat A (1989). Honey--a remedy rediscovered. J R Soc Med.

[119] Mandal MD, Mandal S (2011). Honey: its medicinal property and antibacterial activity. Asian Pac J Trop Biomed.

[120] Maghsoudi H, Moradi S (2015). Honey: a skin graft fixator convenient for both patient and surgeon. Indian J Surg.

[121] Subrahmanyam M (2015). Honey dressing accelerates split-thickness skin graft donor site healing. Indian J Surg.

[122] Oryan A, Alemzadeh E, Moshiri A (2016). Biological properties and therapeutic activities of honey in wound healing: a narrative review and meta-analysis. J Tissue Viability.

[123] Schramm DD, Karim M, Schrader HR, Holt RR, Cardetti M, Keen CL (2003). Honey with high levels of antioxidants can provide protection to healthy human subjects. J Agric Food Chem.

[124] Bashkaran K, Zunaina E, Bakiah S, Sulaiman SA, Sirajudeen K, Naik V (2011). Anti-inflammatory and antioxidant effects of Tualang honey in alkali injury on the eyes of rabbits: experimental animal study. BMC Complement Altern Med.

[125] Attanzio A, Tesoriere L, Allegra M, Livrea MA (2016). Monofloral honeys by Sicilian black honeybee (Apis mellifera ssp. sicula) have high reducing power and antioxidant capacity. Heliyon.

[126] Rzepecka-Stojko A, Stojko J, Kurek-Górecka A (2015). Polyphenols from bee pollen: structure, absorption, metabolism and biological activity. Molecules.

[127] Sun LP, Shi FF, Zhang WW, Zhang ZH, Wang K (2020). Antioxidant and anti-inflammatory activities of safflower (Carthamus tinctorius L.) honey extract. Foods.

[128] Ranneh Y, Akim AM, Hamid HA (2021). Honey and its nutritional and anti-inflammatory value. BMC Complement Med Ther.

[129] Zbuchea A (2014). Up-to-date use of honey for burns treatment. Ann Burns Fire Disasters.

[130] Majtán J, Kovácová E, Bíliková K, Simúth J (2006). The immunostimulatory effect of the recombinant apalbumin 1-major honeybee royal jelly protein-on TNFalpha release. Int Immunopharmacol.

[131] Bazaid AS, Aldarhami A, Gattan H, Aljuhani B (2021). Saudi honey: a promising therapeutic agent for treating wound infections. Cureus.

[132] Yaghoobi R, Kazerouni A, Kazerouni O (2013). Evidence for clinical use of honey in wound healing as an anti-bacterial, anti-inflammatory anti-oxidant and anti-viral agent: a review. Jundishapur J Nat Pharm Prod.

[133] Sartore S, Boyd S, Slabaugh D (2021). Honey and its antimicrobial properties: a function of a single component, or the sum of its parts?. Cureus.

[134] Nolan VC, Harrison J, Cox JA (2019). Dissecting the antimicrobial composition of honey. Antibiotics (Basel).

[135] Albaridi NA (2019). Antibacterial potency of honey. Int J Microbiol.

[136] Scepankova H, Combarros-Fuertes P, Fresno JM (2021). Role of honey in advanced wound care. Molecules.

[137] Gośliński M, Nowak D, Kłębukowska L (2020). Antioxidant properties and antimicrobial activity of manuka honey versus Polish honeys. J Food Sci Technol.

[138] Molan PC (2001). Potential of honey in the treatment of wounds and burns. Am J Clin Dermatol.

[139] Martinotti S, Ranzato E (2018). Honey, wound repair and regenerative medicine. J Funct Biomater.

[140] Martinotti S, Bucekova M, Majtan J, Ranzato E (2019). Honey: an effective regenerative medicine product in wound management. Curr Med Chem.

[141] Hixon KR, Klein RC, Eberlin CT, Linder HR, Ona WJ, Gonzalez H, Sell SA (2019). A critical review and perspective of honey in tissue engineering and clinical wound healing. Adv Wound Care (New Rochelle).

[142] Giretzlehner M, Dirnberger J, Owen R, Haller HL, Lumenta DB, Kamolz LP (2013). The determination of total burn surface area: how much difference?. Burns.

[143] Çomçalı B, Ceylan C, Altun Özdemir B, Ocaklı S, Pehlevan Özel H, Çınar Yastı A (2022). Seasonal effects on the mechanisms of burn injuries. Turk J Surg.

[144] Thom D (2017). Appraising current methods for preclinical calculation of burn size - a pre-hospital perspective. Burns.

[145] Bourgi J, Yaacoob E, Berberi M, Chedid M, Sfeir P, Yaacoub C, Ghanime G (2019). Factors affecting length of stay among pediatric and adult patients admitted to the Lebanese Burn Centre: a retrospective study. Ann Burns Fire Disasters.

[146] Holland AJ (2006). Pediatric burns: the forgotten trauma of childhood. Can J Surg.

[147] Bresler RM, Barksdale E, Hansen EN (2022). Pediatric burn care in the developing world: where are the gaps in research and what can be done?. J Burn Care Res.

[148] Purcell LN, Banda W, Williams B, Gallaher J, Charles A (2020). The effect of surgical intervention on pediatric burn injury survival in a resource-poor setting. J Surg Res.

[149] Mukagaju F, Velin L, Miranda E (2021). What is known about burns in East Africa? a scoping review. J Surg Res.

[150] Nunez Lopez O, Cambiaso-Daniel J, Branski LK, Norbury WB, Herndon DN (2017). Predicting and managing sepsis in burn patients: current perspectives. Ther Clin Risk Manag.

[151] Torres MJ, Peterson JM, Wolf SE (2021). Detection of infection and sepsis in burns. Surg Infect (Larchmt).

[152] Spelten O, Wetsch WA, Braunecker S, Genzwürker H, Hinkelbein J (2011). Estimation of substitution volume after burn trauma: systematic review of published formulae (Article in German). Anaesthesist.

[153] Rode H, Rogers AD, Cox SG, Allorto NL, Stefani F, Bosco A, Greenhalgh DG (2014). Burn resuscitation on the African continent. Burns.

[154] Al-Benna S (2011). Fluid resuscitation protocols for burn patients at intensive care units of the United Kingdom and Ireland. Ger Med Sci.

[155] Ete G, Chaturvedi G, Barreto E, Paul M K (2019). Effectiveness of Parkland formula in the estimation of resuscitation fluid volume in adult thermal burns. Chin J Traumatol.

[156] Abedin M, Rahman FN, Rakhshanda S, Mashreky SR, Rahman AK, Hossain A (2022). Epidemiology of non-fatal burn injuries in children: evidence from Bangladesh Health and Injury Survey 2016. BMJ Paediatr Open.

[157] Agbenorku P, Agbenorku M, Fiifi-Yankson PK (2013). Pediatric burns mortality risk factors in a developing country’s tertiary burns intensive care unit. Int J Burns Trauma.

[158] Jeschke MG, Mlcak RP, Finnerty CC (2008). Gender differences in pediatric burn patients: does it make a difference?. Ann Surg.

[159] Pelizzo G, Lanfranchi G, Pantaloni M (2022). Epidemiological and clinical profile of pediatric burns in the COVID-19 era: the experience of a reference center. Children (Basel).

[160] Pompermaier L, Elmasry M, Abdelrahman I, Fredrikson M, Sjöberg F, Steinvall I (2018). Are there any differences in the provided burn care between men and women? a retrospective study. Burns Trauma.

[161] Purcell LN, Yohann A, Banda W, Gallaher J, Charles A (2021). Sex dimorphism in pediatric burn mortality in Malawi: a propensity matched analysis. Burns.

[162] Tresh A, Baradaran N, Gaither TW (2018). Genital burns in the United States: disproportionate prevalence in the pediatric population. Burns.

[163] Mehta K, Gyedu A, Otupiri E, Donkor P, Mock C, Stewart B (2021). Incidence of childhood burn injuries and modifiable household risk factors in rural Ghana: a cluster-randomized, population-based, household survey. Burns.

[164] Hagstrom M, Wirth GA, Evans GR, Ikeda CJ (2003). A review of emergency department fluid resuscitation of burn patients transferred to a regional, verified burn center. Ann Plast Surg.

[165] Klein MB, Nathens AB, Emerson D, Heimbach DM, Gibran NS (2007). An analysis of the long-distance transport of burn patients to a regional burn center. J Burn Care Res.

[166] Samuel JC, Campbell EL, Mjuweni S, Muyco AP, Cairns BA, Charles AG (2011). The epidemiology, management, outcomes and areas for improvement of burn care in central Malawi: an observational study. J Int Med Res.

[167] Palmer JH, Sutherland AB (1987). Problems associated with transfer of patients to a regional burns unit. Injury.

[168] Curtis EE, Yenikomshian HA, Carrougher GJ, Gibran NS, Mandell SP (2020). Early patient deaths after transfer to a regional burn center. Burns.

[169] Ibrahim SB, Omar MB, Gan EC, Rauf A, Johari NB, Yusof HB (1995). Childhood burns at the Paediatric Institute Kuala Lumpur. Med J Malaysia.

[170] Romanowski KS, Palmieri TL (2017). Pediatric burn resuscitation: past, present, and future. Burns Trauma.

[171] Chakraborty RK, Burns B (2022). Systemic inflammatory response syndrome. https://www.ncbi.nlm.nih.gov/books/NBK547669/.

[172] Aziz Z, Abdul Rasool Hassan B (2017). The effects of honey compared to silver sulfadiazine for the treatment of burns: a systematic review of randomized controlled trials. Burns.

[173] Malik KI, Malik MA, Aslam A (2010). Honey compared with silver sulphadiazine in the treatment of superficial partial-thickness burns. Int Wound J.

[174] Nagane NS, Ganu JV, Bhagwat VR, Subramanium M (2004). Efficacy of topical honey therapy against silver sulphadiazine treatment in burns: a biochemical study. Indian J Clin Biochem.

[175] Osman S, Umar H, Hashmi Y, Jawaid A, Ahmed Z (2022). The efficacy of honey compared to silver sulfadiazine for burn wound dressing in superficial and partial thickness burns—a systematic review and meta-analysis. Trauma Care.

[176] Khoo YT, Halim AS, Singh KK, Mohamad NA (2010). Wound contraction effects and antibacterial properties of Tualang honey on full-thickness burn wounds in rats in comparison to hydrofibre. BMC Complement Altern Med.

[177] Hadinata CB, Arif A, Irfannuddin Irfannuddin (2020). Comparison of honey effectiveness with silver sulfadiazine 1% on the formation of epithelial post deep dermal burns injury at rats. Majalah Kedokteran Sriwijaya.

[178] Gupta SS, Singh O, Bhagel PS, Moses S, Shukla S, Mathur RK (2011). Honey dressing versus silver sulfadiazene dressing for wound healing in burn patients: a retrospective study. J Cutan Aesthet Surg.

[179] Baghel PS, Shukla S, Mathur RK, Randa R (2009). A comparative study to evaluate the effect of honey dressing and silver sulfadiazene dressing on wound healing in burn patients. Indian J Plast Surg.

[180] Rani M, Schwacha MG (2012). Aging and the pathogenic response to burn. Aging Dis.

[181] Savetamal A (2021). Infection in elderly burn patients: what do we know?. Surg Infect (Larchmt).

[182] Stanojcic M, Chen P, Xiu F, Jeschke MG (2016). Impaired immune response in elderly burn patients: new insights into the immune-senescence phenotype. Ann Surg.

[183] Emsen IM (2007). A different and safe method of split thickness skin graft fixation: medical honey application. Burns.

